# Evolution of acute hepatitis C virus infection in a large European city: Trends and new patterns

**DOI:** 10.1371/journal.pone.0187893

**Published:** 2017-11-14

**Authors:** César Garriga, Sandra Manzanares-Laya, Patricia García de Olalla, Pilar Gorrindo, Sabela Lens, Ricard Solà, María Martínez-Rebollar, Montserrat Laguno, Jordi Navarro, Xavier Torras, Mercè Gurguí, María-Jesús Barberá, Josep Quer, Eva Masdeu, Pere Simón, Miriam Ros, Anna de Andrés, Joan A. Caylà

**Affiliations:** 1 Epidemiology Service, Public Health Agency of Barcelona, Barcelona, Spain; 2 Spanish Field Epidemiology Training Programme (FETP/PEAC), National Centre for Epidemiology, Instituto de Salud Carlos III, Madrid, Spain; 3 CIBER de Epidemiología y Salud Pública (CIBERESP), Madrid, Spain; 4 Liver Unit, Hospital Clínic, Barcelona. IDIBAPS, Barcelona, Spain; 5 CIBER of Hepatic and Digestive Diseases (CIBERehd), Madrid, Spain; 6 Internal Medicine-Infectious Diseases, Hospital del Mar, Barcelona, Spain; 7 Hospital Clinic- August Pi i Sunyer Biomedical Research Institute, University of Barcelona, Barcelona, Spain; 8 Infectious Diseases, Hospital Vall de Hebron, Universitat Autònoma de Barcelona, Barcelona, Spain; 9 Department of Gastroenterology, Hospital de la Santa Creu i Sant Pau, CIBERehd, Barcelona, Spain; 10 Infectious Diseases Unit, Hospital de la Santa Creu i Sant Pau, Universitat Autònoma de Barcelona, Barcelona, Spain; 11 Sexually Transmitted Infections Unit, Hospital Vall de Hebron, Universitat Autònoma de Barcelona, Barcelona, Spain; 12 Liver Unit, Internal Medicine, Laboratory of Malalties Hepàtiques, Vall d'Hebron Institut de Recerca-Hospital Universitari Vall d´Hebron, (VHIR-HUVH), Universitat Autònoma de Barcelona, Barcelona, Spain; University of Cincinnati College of Medicine, UNITED STATES

## Abstract

The aims of this study were to describe the evolution of acute hepatitis C virus (HCV) infections since 2004 and to determine its associated factors. Acute HCV infections diagnosed in Barcelona from 2004 to 2015 were included. Incidence ratios (IR) were then estimated for sex and age groups. Cases were grouped between 2004–2005, 2006–2011 and 2012–2015, and their incidence rate ratios (IRR) were calculated. In addition, risk factors for acute HCV infection were identified using multinomial logistic regression for complete, available and multiple imputed data. 204 new HCV cases were identified. Two peaks of higher IR of acute HCV infection in 2005 and 2013 were observed. Men and those aged 35–54 had higher IR. IRR for men was 2.9 times greater than in women (95% confidence intervals (CI): 1.8 ‒ 4.7). Factors related to the period 2012–2015 (versus 2006–2011) were: a) sexual risk factor for transmission versus nosocomial (relative-risk ratio (RRR): 13.0; 95% CI: 2.3 ‒ 72.1), b) higher educated versus lower (RRR: 5.4; 95% CI: 1.6 ‒ 18.7), and c) HIV co-infected versus not HIV-infected (RRR: 53.1; 95% CI: 5.7 ‒ 492.6). This is one of the few studies showing IR and RRRs of acute HCV infections and the first focused on a large city in Spain. Sexual risk for transmission between men, higher educational level and HIV co-infection are important factors for understanding current HCV epidemic. There has been a partial shift in the pattern of the risk factor for transmission from nosocomial to sexual.

## Introduction

The disease burden of hepatitis C virus (HCV) infection is high, with more than 185 million people estimated to be infected worldwide [1]. HCV infection estimates for the Spanish population range between 480,000 and 760,000 people[2].

Transmission may occur between injection drug users (IDU) sharing contaminated needles as well as in health caring settings as a result of reuse or ineffective sterilisation of medical equipment[3]. Occasionally, reported cases are associated with contaminated material used for tattooing, piercing and acupuncture[4]. In turn, sexual transmission of HCV has become more important in some large cities[5]. However, due to the usually insidious nature of the acute infection, most of the time the method of transmission remains unclear [6]. This issue has been addressed after implementation of mandatory surveillance of HCV infections in the European Union[7].

Historically, acute HCV infections in clinical settings have been associated with the reuse of needles, accidental needlestick injuries, haemodialysis devices and liver or other solid transplantations [8]. Implementation of screening programs limited HCV transmission mainly to IDU, during the 80’s and 90’s; and immigrants from endemic countries [9]. Additionally, higher prevalence of HCV infection has been observed in prisons [10]. Transmission can also occur between people living together and, very rarely, by vertical transmission.

Heterosexual intercourse is not considered an important route of transmission. However, since the mid-2000s outbreaks of sexual transmission of HCV has been reported among men who have sex with men (MSM) in large cities across Europe, USA and Australia[11–13].This increase is higher in MSM co-infected with human immunodeficiency virus (HIV). Indeed, a recent systematic review has shown that HIV-positive MSM were at higher risk for acute HCV infection than HIV-negative MSM[5]. The cause for this difference is yet to be determined, as it is not clear whether it is due to a higher consumption of injected drugs that goes unreported, to a higher susceptibility or to other sexual practices that pose a higher risk for HCV acquisition amongst HIV-positive MSM.

A comprehensive epidemiological survey is presented here of acute HCV infection in Barcelona, Spain, from 2004 to 2015 to describe incidence and trends and to identify risk factors associated with acute HCV infection in different periods using the registry of mandatory notifiable diseases of the city.

## Materials and methods

### Study design, setting and cases

A descriptive design was carried out for all acute HCV cases detected by the surveillance system in the city of Barcelona. HCV infection started to be recorded separately from other types of viral hepatitis in 2004, since markers differentiating hepatitis C from other types of hepatitis different than hepatitis A and B were not available before then. Previously, they were notified as “other viral hepatitis other than A or B”, and they were notified since the early eighties. Therefore, acute HCV cases living in Barcelona from 2004 to 2015 are described. Acute HCV cases were defined as having a positive anti-HCV or ribonucleic acid of HCV detected using polymerase chain reaction (PCR-RNA) test after a negative test in the previous six months[14], that could show up as symptomatic (jaundice, anorexia, abdominal discomfort, dark urine…), or asymptomatic. In addition, cases with laboratory confirmation who did not have any previous test done or with a previous negative test of more than 6 months were also included if they increased transaminases more than 10 times their normal values or they had had a recent contact with a HCV case confirmed. Trained public health nurses interviewed the cases ascertaining self-reported risk factors using an epidemiological survey especially designed for hepatitis C. They completed the information with physicians’ feedback, laboratory data and/or revision of medical histories. The Barcelona HCV surveillance system is an active system that collects data provided by doctors, electronic notifications and hospital discharges regarding patients diagnosed with HCV. Public and private laboratories are also part of the surveillance system. Professionals of the microbiological laboratories from health centres report notifiable diseases and outbreaks to the surveillance system. In addition, public health nurses of our team requested and searched actively for incomplete laboratory data.

### Data collection

Clinicians completed a standard data collection form and used nominal identification. Socio-demographic and laboratory data were also collected including HIV co-infection.

Date of symptoms onset was taken as the reference date for start of disease. However, if this date was missing due to lack of symptoms, the date of diagnosis, or notification in its absence, was used as the reference date. Cases were classified as isolated or as associated with other cases, thus being part of an outbreak. In addition, information about the possible risk of exposure in the previous 6 months was recorded and it was categorised into the following risk groups: sexual, nosocomial (surgery, invasive medical exploration, contact with dialysis machines, kidney transplant, transfusion reception, blood contact, and maxillofacial intervention), IDU, and others (acupuncture, tattoo, piercing…). Sexual risk factor was ascertained from cases involved in an outbreak investigation of sexual transmission of HCV; among men reporting to have had sex with men, and those living with their sexual partner with HCV infection. Finally, details about follow-up and outcome were collected.

Population data for the city was taken from Barcelona City Council Statistics department including the overall population of the city, stratified by age groups and sex for calendar year[15]. District household income information was obtained from the Barcelona council website[16]. This is an index calculated from (a) educational level of the population, (b) work status, (c) the number of cars, (d) the horsepower of newly bought cars, and (e) second-hand housing prices. Lastly, a double-check for HIV co-infection was done using the HIV Registry.

### Statistical analysis

Cases were grouped in three periods according to the incidence trend observed: 2004–2005; 2006–2011 and 2012–2015. The following variables were compared among periods: sex; age; origin (Spanish-born versus foreign born); educational level (illiterate/primary/lower secondary and upper secondary/university); district household income at diagnosis, which is measured as standard units (low-medium [<100], high [100–159] and very high [>159])[16, 17]; risk factor for HCV infection (nosocomial, sexual[almost exclusively among MSM][5, 18], IDU, and other low risk factor[acupuncture, tattoo, piercing, wound]); and HIV sero-status.

Differences between periods were evaluated using χ^2^ test or Fisher’s exact test for categorical variables. Age between periods was evaluated using Student's t-test.

#### Trend analysis

A trend analysis was performed to estimate the incidence ratio (IR) of acute HCV infections per 100,000 inhabitants by calendar year, overall and according to sex and age. Subsequently ratios for men/women were obtained with their 95% confidence intervals (CI).

Period incidence rate ratios (IRR) were calculated using negative binomial regression, taking the 2006–2011 as reference because as it represents the steadiest period. The model was adjusted by age group and sex. The associated CI were obtained using a negative binomial distribution.

#### Multiple imputation

Missing values were common for risk factors for transmission before 2012. Multiple imputation by chained equations was employed, which accounts for the uncertainty around missing data [19]. Then, 100 imputed datasets were created. Imputed values were generated taking into account the other risk factors that an individual may have relative to the entire dataset. Thus, all the patients were included in the multivariable analysis. Details providing information about the suitability of the missing data for multiple imputation are provided in the appendix (S1 Appendix).

#### Multivariable analysis

A multinomial logistic regression model was fitted to identify risk factors for acute HCV diagnosis associated with the periods 2004–2005 and 2012–2015, taking 2006–2011 as the reference (the steadiest period). The adjusted relative-risk ratio (RRR) and its 95% CI was the measure of association. Variables were considered as candidates for inclusion in the multivariable model if the associated univariable *P*-value was <0.30 [20, 21]. A backward procedure based on the Wald test was then used to make a first selection of significant variables. Age was used in the models as a continuous variable made up of 5-year age groups. Finally, models were adjusted for any confounding factors (i.e., sex, age and origin) and statistically significant factors from the univariable model. Model fit was checked using Hosmer-Lemeshow test of goodness of fit. Risk factors were selected for *P*-values<0.05.

The multivariable analysis was done under three approaches: i) complete case model, which not included individuals with missing data for any of the risk factors; ii) available case model, which included all subjects, missing data were analysed as another category for educational level, district household income and risk factors for HCV transmission; and iii) imputed model, which included all subjects, missing values were imputed.

Analyses were performed using SPSS v.22.0 (Chicago, IL) and Stata v13 (College Station, TX).

### Ethical considerations

Acute HCV infection is of mandatory notification for health professionals in compliance with the law 203/2015 (15th Sep 2015) of the Health Department of Generalitat de Catalunya. The Public Health Agency of Barcelona is the Health Authority for the surveillance and control of the data routinely collected on communicable diseases under mandatory notification as well as on outbreaks in the city of Barcelona. The data source where are these data is the Mandatory Communicable Disease Registry. Data were treated in a strictly confidential manner according to the ethical principles of the Helsinki Declaration [22] and Law 15/1999 of Personal Data Protection in Spain[23]. Patient information was anonymized and de-identified prior to analysis (S2 Appendix Dataset).

## Results

A total of 428 HCV infections were reported and 204 (47.7%) of them were acute infections. According to the three study periods, there were 53 (26.0%) cases from 2004 to 2005, 57 (27.9%) from 2006 to 2011, and 94 (46.1%) from 2012 to 2015. The mean age of patients was 45 years (standard deviation ±14 years) ranging between 3 and 90 years.

Characteristics of the patients included are summarized for each of the three time periods in Table 1. A higher proportion of men, foreign-born, people having completed upper secondary or university studies, and high income according to the district was found in the 2012–2015 period in comparison with the other periods of the study. Additionally, there were more cases with sexual risk factor and higher proportion of HIV-infected individuals among men, while nosocomial was the most important risk factor for women in the three periods. Indeed, there were only 3 cases of heterosexual risk factor in women.

**Table 1:** Characteristics of acute cases of hepatitis C infection according to period (total, men, and women). Barcelona city (2004–2015).

	**Total (n = 204)**								
	**Total**		**2004–2005**		**2006–2011**		**2012–2015**		
	**n**		**n**		**n**		**n**		***P* value**
**Sex (%)**									<0.01
Men	**151**	(74.0)	**29**	(54.7)	**39**	(68.4)	**83**	(88.3)	
Women	**53**	(26.0)	**24**	(45.3)	**18**	(31.6)	**11**	(11.7)	
**Median age, years (SD)**^**a**^	**204**	45 (±14)	**53**	48 (±15)	**57**	47 (±17)	**94**	43 (±11)	0.72^c^ 0.16^d^
**Country of origin (%)**									0.10
Spanish-born	**135**	(66.2)	**38**	(71.7)	**42**	(73.7)	**55**	(58.5)	
Foreign born	**69**	(33.8)	**15**	(28.3)	**15**	(26.3)	**39**	(41.5)	
**Education level completed (%)**									<0.01
Illiteracy/Primary/Lower secondary	**81**	(39.8)	**30**	(56.6)	**35**	(61.4)	**16**	(17.0)	
Upper secondary/University	**87**	(42.6)	**12**	(22.6)	**15**	(26.3)	**60**	(63.8)	
Missing	**36**	(17.6)	**11**	(20.8)	**7**	(12.3)	**18**	(19.1)	
**District household income**^**a**^^,^ ^**b**^ **(%)**									<0.01^e^
Low-medium	**129**	(63.2)	**34**	(64.2)	**46**	(80.7)	**49**	(52.1)	
High	**59**	(28.9)	**12**	(22.6)	**7**	(12.3)	**40**	(42.6)	
Very high	**12**	(5.9)	**3**	(5.7)	**4**	(7.0)	**5**	(5.3)	
Missing	**4**	(2.0)	**4**	(7.5)	**0**	**―**	**0**	**―**	
**Risk factor for HCV transmission(%)**									<0.01^e^
Sexual	**74**	(36.3)	**1**	(1.9)	**4**	(7.0)	**69**	(73.4)	
Nosocomial	**48**	(23.5)	**13**	(24.5)	**22**	(38.6)	**13**	(13.8)	
IDU	**14**	(6.9)	**1**	(1.9)	**9**	(15.8)	**4**	(4.3)	
Others	**7**	(3.4)	**3**	(5.7)	**1**	(1.8)	**3**	(3.2)	
Missing	**61**^g^	(29.9)	**35**	(66.0)	**21**	(36.8)	**5**	(5.3)	
**HIV status**^**a**^ **(%)**									<0.01
Positive	**66**	(32.4)	**1**	(1.9)	**1**	(1.8)	**64**	(68.1)	
Negative^**f**^	**138**	(67.6)	**52**	(98.1)	**56**	(98.2)	**30**	(31.9)	
	**Men (n = 151)**								
	**Total**		**2004–2005**		**2006–2011**		**2012–2015**		
	**n**		**n**		**n**		**n**		***P* value**
**Median age, years (SD)**^**a**^	**151**	44 (±13)	**29**	48 (±15)	**39**	46 (±15)	**83**	42 (±10)	0.58^c^ 0.81^d^
**Country of origin (%)**									0.41
Spanish-born	**91**	(60.3)	**17**	(58.6)	**27**	(69.2)	**47**	(56.6)	
Foreign born	**60**	(39.7)	**12**	(41.4)	**12**	(30.8)	**36**	(43.4)	
**Education level completed (%)**									<0.01
Illiteracy/Primary/Lower secondary	**50**	(33.1)	**17**	(58.6)	**22**	(56.4)	**11**	(13.3)	
Upper secondary/University	**73**	(48.3)	**6**	(20.7)	**10**	(25.6)	**57**	(68.7)	
Missing	**28**	(18.5)	**6**	(20.7)	**7**	(17.9)	**15**	(18.1)	
**District household income**^**b**^ **(%)**									<0.01^e^
Low-medium	**90**	(59.6)	**20**	(69.0)	**31**	(79.5)	**39**	(47.0)	
High	**51**	(33.8)	**5**	(17.2)	**7**	(17.9)	**39**	(47.0)	
Very high	**7**	(4.6)	**1**	(3.4)	**1**	(2.6)	**5**	(6.0)	
Missing	**3**	(2.0)	**3**	(10.3)	**0**	**―**	**0**	**―**	
**Risk factor for HCV transmission(%)**									<0.01^e^
Sexual	**71**	(47.0)	**0**	**―**	**3**	(7.7)	**68**	(81.9)	
Nosocomial	**25**	(16.6)	**7**	(24.1)	**12**	(30.8)	**6**	(7.2)	
IDU	**11**	(7.3)	**1**	(3.4)	**7**	(17.9)	**3**	(3.6)	
Others	**6**	(4.0)	**3**	(10.3)	**1**	(2.6)	**2**	(2.4)	
Missing	**38**	(25.2)	**18**	(68.1)	**16**	(41.0)	**4**	(4.8)	
**HIV status**^**a**^ **(%)**									<0.01
Positive	**64**	(42.4)	**1**	(3.4)	**0**	**―**	**63**	(75.9)	
Negative^**f**^	**87**	(57.6)	**28**	(96.6)	**39**	(100.0)	**20**	(24.1)	
	**Women (n = 53)**								
	**Total**		**2004–2005**		**2006–2011**		**2012–2015**		
	**n**		**n**		**n**		**n**		***P* value**
**Median age, years (SD)**^**a**^	**53**	50 (±17)	**24**	48 (±16)	**18**	50 (±20)	**11**	55 (±17)	0.16^c^ 0.48^d^
**Country of origin (%)**									0.57^e^
Spanish-born	**44**	(83.0)	**21**	(87.5)	**15**	(83.3)	**8**	(72.7)	
Foreign born	**9**	(17.0)	**3**	(12.5)	**3**	(16.7)	**3**	(27.3)	
**Education level completed (%)**									0.19^e^
Illiteracy/Primary/Lower secondary	**31**	(58.5)	**13**	(54.2)	**13**	(72.2)	**5**	(45.5)	
Upper secondary/University	**14**	(26.4)	**6**	(25.0)	**5**	(27.8)	**3**	(27.3)	
Missing	**8**	(15.1)	**5**	(20.8)	**0**	**―**	**3**	(27.3)	
**District household income**^**b**^ **(%)**									0.05^e^
Low-medium	**39**	(73.6)	**14**	(58.3)	**15**	(83.3)	**10**	(90.9)	
High	**8**	(15.1)	**7**	(29.2)	**0**	**―**	**1**	(9.1)	
Very high	**5**	(9.4)	**2**	(8.3)	**3**	(16.7)	**0**	**―**	
Missing	**1**	(1.9)	**1**	(4.2)	**0**	**―**	**0**	**―**	
**Risk factor for HCV transmission(%)**									<0.01^e^
Sexual	**3**	(5.7)	**1**	(4.2)	**1**	(5.6)	**1**	(9.1)	
Nosocomial	**23**	(43.4)	**6**	(25.0)	**10**	(55.6)	**7**	(63.6)	
IDU	**3**	(5.7)	**0**	**―**	**2**	(11.1)	**1**	(9.1)	
Others	**1**	(1.9)	**0**	**―**	**0**	**―**	**1**	(9.1)	
Missing^g^	**23**	(43.4)	**17**	(70.8)	**5**	(27.8)	**1**	(9.1)	
**HIV status**^**a**^ **(%)**									0.30^e^
Positive	**2**	(3.8)	**0**	**―**	**1**	(5.6)	**1**	(9.1)	
Negative^**f**^	**51**	(96.2)	**24**	(100.0)	**17**	(94.4)	**10**	(90.9)	

Hepatitis C virus, HCV; standard deviation, SD; injective drug user, IDU; human immunodeficiency virus, HIV

^a^ At the time of HCV diagnosis.

^b^ 2013 Barcelona household income according to district where the person diagnosed of HCV lived.

^c^ 2-tailed significant level of t-test for equality of means between 2004–2005 and 2006–2011 periods.

^d^ 2-tailed significant level of t-test for equality of means between 2006–2011 and 2012–2014 periods.

^e^ Significant level of 2-sided Fisher's exact statistic.

^f^ Since 2011 HIV reporting became mandatory.

^g^ Of them, 38 (62.3%) were men, 46 (75.4%) were Spanish-born, 36 (59.0%) were illiterate or had only completed primary/lower secondary studies, 44 (72.1%) lived in low/medium income districts, and none of them were HIV-infected.

**Fig 1:**
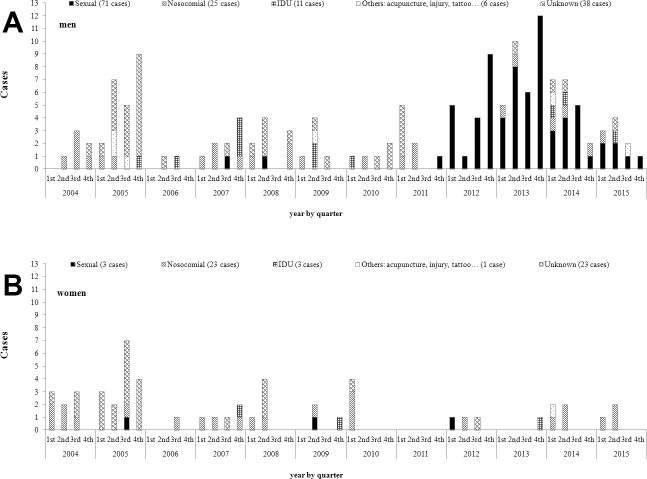
Cases of acute hepatitis C by symptom onset date grouped by quarter and risk factor. **Barcelona city, 2004─ 2015.** (A) Men. (B) Women. Diagnosis date was used instead of missing symptom onset dates in 52 cases, notification date was used instead of missing symptom onset and diagnosis dates in 52 cases. Mean months elapsed among symptom onset date and diagnosis date was 1.5 (standard deviation (sd) = 8.0; n = 77); mean months elapsed among diagnosis date and notification date was 2.5 (sd = 5.3; n = 129). Hepatitis C virus, HCV; incidence ratio, IR; years old, y.

Risk factors changed along the study. Nosocomial or unknown way were the more prevalent groups in 2004–2005 and 2006–2011 (n = 13; 24% and n = 35; 66%; and n = 22; 38.6% and n = 21; 31.6%, respectively). In the 2012–2015 period, sexual risk factor included the majority of new cases (n = 69; 73.4%). According to gender, this increase was observed in men since 2012, but not in women (Fig 1).

Because cases before 2012 were searched for retrospectively we did not have information about risk factors for HCV infection for 56 (50.9%) individuals between 2004 and 2011, the period during which this variable was not systematically recorded. Thus, we imputed 61 missing values for risk factors, as well as 36 ones for educational level completed and 4 for district household income.

During the whole study period there were 28 (13.7%) cases living with their sexual partners also with HCV infection, 25 of them were men and 20 of these men had HIV co-infection. On the other hand, 5 (2.5%) cases were cohabiting with HCV infected individuals who were not their sexual partners. Most of cases with sexual risk for transmission were considered to be part of an outbreak of sexual transmission of hepatitis C (63 out of 71 males, 89%). Health-care related infections reported were: surgeries (27 cases, 13.2% of all acute HCV cases reported), invasive medical exploration (17; 8.3%), dialysis (12; 5.9%), kidney transplantation (9; 4.4%), blood derivate transfusion (9; 4.4%), blood contact (6; 2.9%), and maxillofacial intervention (3; 1.5%).

**Fig 2:**
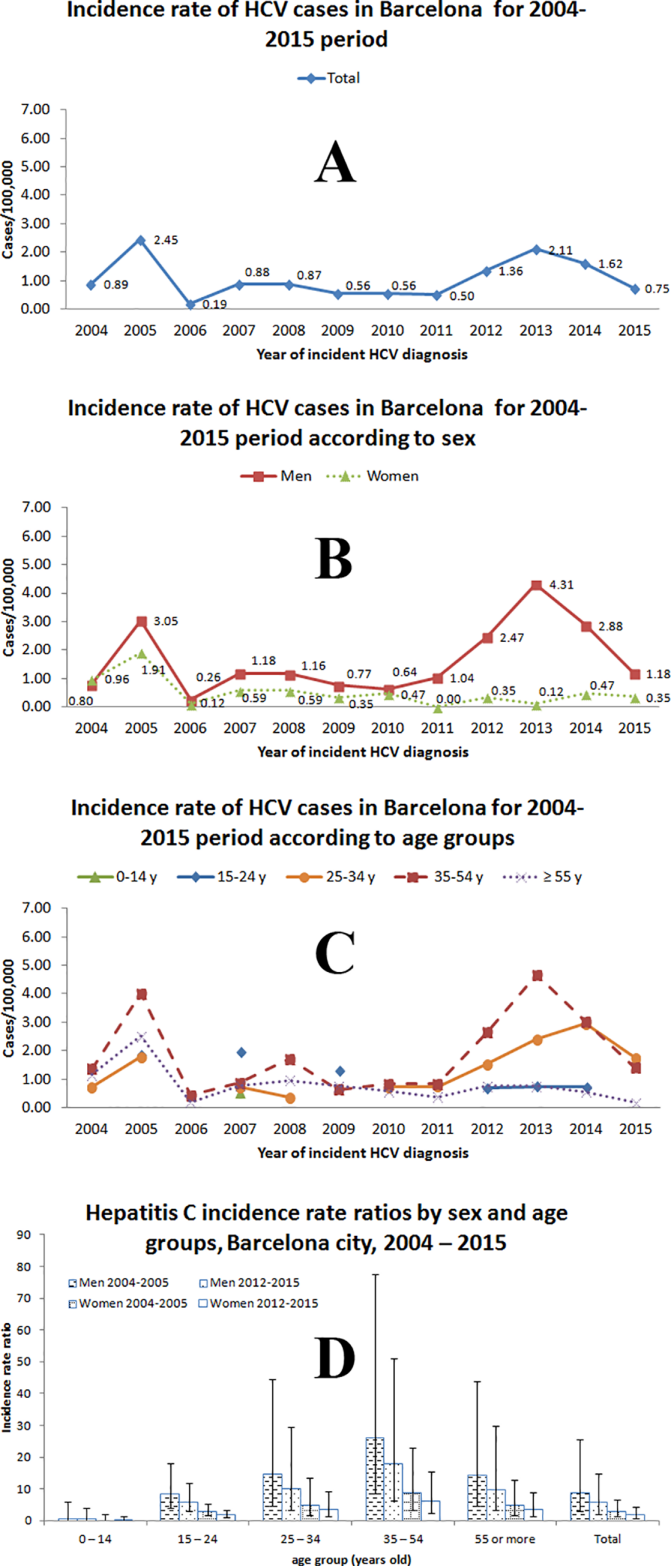
Trend in number of acute hepatitis C cases per 100,000 inhabitants and incidence rate ratio of hepatitis C by sex and age, Barcelona city residents, 2004–2015. (A) IR for total cases. (B) IR for men and women. (C) IR layered by age group, data label is omitted in this panel to facilitate its comprehension. (D) Incidence rate ratio, reference period is 2006–2011. See explanation of dates used in the footnote of the Fig 1.

There were two peaks of higher IR of acute HCV infection in 2005 and 2013, with 2.45 cases/100,000 inhabitants and 2.11, respectively (Fig 2A). Incidence rate was higher among men during the study, with similar trends between men and women from 2004 to 2010 (Fig 2B). Since 2011, the IR of the male group increased substantially while that of women remained stable. The ratio for men/women was 1.3 (95% CI: 0.8–2.3), 2.4 (95% CI: 1.4–4.2) and 8.4 (95% CI: 4.5–15.7) for the periods 2004–2005, 2006–2011 and 2012–2015, respectively. Lastly, according to age group, the highest IR was for 35–54 years individuals (Fig 2C).

The overall IRR of acute HCV infection was 92% lower in the youngest group (0 ‒ 14 years; IRR: 0.1; 95% CI: <0.1 ‒ 0.6) compared to the 15‒ 24 years group, whereas the overall IRR for the group of 35 ‒ 54 years was 3.0 times higher (95% CI: 1.4 ‒ 6.5). The overall IRR in men was 2.9 (95% CI: 1.8 ‒ 4.7) times greater than in women. Taking 2006–2011 as reference, we observed higher IRR in the two epidemic waves in 2004–2005 and 2012–2015 for men and women over 14 years (Fig 2D).

There were no differences in the relative risk for suffering acute HCV infection between periods 2006–2011 and 2004–2005 (S1 Table). Nevertheless, there were increased RRR for the following factors related to the period 2012–2015 compared to 2006–2011: a) sexual risk factor versus nosocomial, b) higher education versus lower, and c) HIV co-infected versus not HIV-infected (Table 2). The crude analysis showed that being a man and living in high income districts was associated to acute HCV infection diagnosis in period 2012 and 2015.

**Table 2:** Factors associated with acute HCV infection in the period 2012–2015 compared to the period 2006–2011. Complete case model, available case and imputed case. Barcelona city.

		Complete cases model (n = 121)				Available cases model (n = 204)				Imputed model (n = 204)			
		Crude analysis		Adjusted analysis		Crude analysis		Adjusted analysis		Crude analysis		Adjusted analysis	
Variables	Categories	RRR	95% CI	RRR	95% CI	RRR	95% CI	RRR	95% CI	RRR	95% CI	RRR	95% CI
**Sex**	(Women)												
	Men	**6.0**	[2.1–17.2]	0.9	[0.2–3.7]	**3.5**	[1.5–8.1]	0.9	[0.2–3.0]	**3.5**	[1.5–8.1]	0.6	[0.2–2.0]
**Age**^**a**^	(Continuous variable, 5 years groups)	0.9	[0.8–1.1]	**1.4**	[1.0–1.8]	0.9	[0.8–1.0]	**1.4**	[1.1–1.8]	0.9	[0.8–1.0]	1.2	[1.0–1.6]
**Country of origin**	(Spanish–born)												
	Foreign born	**2.8**	[1.1–7.0]	2.7	[0.5–13.4]	2.0	[1.0–4.1]	2.3	[0.5–9.8]	2.0	[1.0–4.1]	1.4	[0.4–5.1]
**Educational level completed**	(Illiteracy/Primary/Lower secondary)												
	Upper secondary/University	**8.3**	[3.3–21.0]	**6.2**	[1.5–25.9]	**8.8**	[3.9–19.8]	**5.9**	[1.6–21.3]	**8.3**	[3.7–18.7]	**5.4**	[1.6–18.7]
	Missing	―	―	―	―	**5.6**	[2.0–16.1]	0.7	[0.1–8.3]	―	―		
**District household income**^**b**^	(Low–medium)												
	High	**6.1**	[1.9–19.4]			**5.4**	[2.2–13.2]			**5.4**	[2.2–13.2]		
	Very high	0.5	[0.1–3.1]			1.2	[0.3–4.6]			1.2	[0.3–4.6]		
	Missing	―	―			1.6	[0.0–]			―	―		
**Risk factor for HCV transmission**	(Nosocomial)												
	Sexual	**38.7**	[10.6–141.9]	**18.5**	[2.7–128.3]	**29.2**	[8.6–98.8]	**23.9**	[3.6–156.4]	**28.0**	[8.5–92.2]	**13.0**	[2.3–72.1]
	IDU	1.1	[0.2–5.5]	1.4	[0.1–17.0]	0.8	[0.2–2.9]	1.2	[0.1–14.2]	0.8	[0.2–3.0]	1.3	[0.2–9.3]
	Others: acupuncture, tattoo. . .	5.3	[0.4–66.2]	19.3	[1.0–388.8]	5.1	[0.5–54.0]	**27.7**	[1.5–517.4]	4.4	[0.5–43.1]	10.9	[0.7–160.1]
	Missing^c^	―	―			0.4	[0.1–1.3]	1.2	[0.3–5.5]	―	―		
**HIV status**^**a**^	(Negative)												
	Positive	**96.0**	[12.2–753.7]	**67.8**	[5.7–807.6]	**119.5**	[15.8–904.5]	**114.3**	[9.5–1369.0]	**119.5**	[15.8–904.5]	**53.1**	[5.7–492.6]

Hepatitis C virus, HCV; relative-risk ratio, RRR; confidence intervals, CI; injective drug user, IDU; and human immunodeficiency virus, HIV. All the models have been adjusted by sex, age, origin, educational level, risk factor for transmission for HCV, and HIV status. Reference categories are shown in brackets. Bold numbers highlight *P*-values<0.05 for Wald test.

^a^ At the time of HCV diagnosis.

^b^ 2013 Barcelona household income according to district where the person diagnosed of HCV lived.

^c^ Of them, 20 (76.9%) were men, 21 (80.7%) were Spanish-born, 15 (57.7%) were illiterate or had only completed primary/lower secondary studies, 21 (80.8%) lived in low/medium income districts, and none of them were HIV-infected.

## Discussion

This study provides a compressive approach of the acute HCV infection epidemic at a population-based level in the city of Barcelona. It takes a long-term view of 12 years to show changes in risk factors, and this crucial information can help inform efficient public health measures. Sexual risk factor has become the principal route of infection in men due to the outbreak of HCV infection in MSM that has been reported in HCV/HIV coinfected subjects [24].

### Key results

This is the first study reporting patterns and trends of acute HCV focusing in a large city. Nosocomial infections in women and sexual practices among men are the two main risk factors of HCV infection in Barcelona. An increase of cases with sexual risk factor in men has been observed since 2012 as the incidence of nosocomial cases decreased. In spite of this, an important number of nosocomial cases have been notified up to 2011. In addition, nosocomial cases would likely have increased if we had determined that unknown risk factor cases were not associated with sexual risk after using multiple imputation method.

### Acute HCV epidemic

Registry of notifiable diseases of Barcelona generates crucial information for acute HCV infection; identifying subpopulations requiring public health interventions; and assessing such efforts. At the level of the city there is only a report from the New York city health department which identified less than 20 acute cases each year[25], likely far below the real number of incident cases. Indeed, cases identified in Barcelona averaged 17 cases each year for a smaller population.

It is assumed cases have previously been underestimated because most acute HCV infections are asymptomatic, e.g. it was estimated only 1 case out of 12.3 in USA were notified in 2011[26]. Underestimation in our context would be lower because several patients were identified to be acute HCV case with no symptoms as a consequence of a previous negative test in the last six months. They were HIV patients that were being tested regularly for HCV.

### Factors associated with increased incident HCV cases between 2012 and 2015

The results demonstrate sexual risk factor, HIV infection, and high educational level are related to an increase of acute HCV cases since 2012.

#### Risk factors for HCV infection

Sexual risk factor has been almost exclusively related to men who are MSM. Indeed, heterosexual transmission of HCV has been demonstrated to have a very low incidence [18]. This is concordant with a global epidemic of sexually transmitted HCV among MSM since 2000´s and probably earlier [27].

Sexual transmission in heterosexual discordant couples is very low [28], but anal sex, fisting and use of sexual toys without condoms or gloves have been linked to a higher risk of transmission [29]. Serosorting has also been associated with a higher frequency of HIV-HCV co-infection[30], and the use of psycho stimulant drugs before and during sexual intercourse can lead to higher risk practices[31]. Eight percent of cases were associated with risk for transmission for sharing injection paraphernalia. Hence, some infections might not be reported for IDUs that are not yet in contact with the health system and those delaying to start treatment for HCV infection in a clinical setting since the time of diagnosis. High incidence reported among IDUs using harm reduction facilities in Barcelona pointed to delaying time to start treatment [32, 33]. Nonetheless, the harm reduction strategies implemented in the city would explain the control of spreading HCV infection in this core population[34–37]. Additionally, the highest population incidence in Spain of heroin use happened in 1980[38]. The peak of injection-related HIV incidence was achieved in 1985[39]. Since 1987 there is an increase in harm reduction coverage due mainly to a decline in drug injection and heroin use (e.g.: in 2010 the opioid substitution treatment was high, 60.3% of the Spanish IDUs)[40].

#### HIV status

The majority of cases with sexual risk factor were men who are probably MSM and most of them were also HIV co-infected. Blood tests monitoring HIV patients led to find many acute HCV cases [24]. This finding is in agreement with other studies [41, 42]. HIV co-infection would facilitate the transmission of the HCV implying greater serum and semen viral loads, and immunodeficiency in mucosa barrier [43].

#### Educational level

Educational level is a proxy for socioeconomic status. Usually a lower educational level is associated with poorer health status and this is also observed for HCV infected people [44]. In fact, a higher percentage of illiteracy, primary and lower secondary educated individuals was seen in the periods 2004–2005 and 2006–2011. Nonetheless this situation changes for the period 2012–2015 when cases with higher educational level were more often affected by acute HCV infection. MSM with sexual risk behaviours often have high educational level [45]. In addition, cases with higher educational level, would more frequently use the Internet and its social networks to achieve occasional sex [45]. Furthermore, MSM affected by sexual diseases have higher educational level than heterosexuals but, among MSM, those with lower educational level have also higher risk getting sexual transmitted infections [46].

### Limitations

The registry of notifiable diseases started to collect information about HCV apart from other hepatitis in 2004, therefore we do not have information from previous years. As a consequence of the nature of the source of the data some valuable information regarding risk factors were not available in the epidemiological survey such as sexual orientation and illicit drug use other than injected. Nevertheless, risk factor for transmission, HIV status and the other risk factors gathered from the epidemiological survey allow us to present a clear and necessary information about the HCV infection in a large city for a wide period not previously reported.

Differences between periods 2006–2011 and 2004–2005 were not found. Therefore, 2005 peak of cases would be due to limitations in the notification of the cases in the first years of the registry.

There were many missing values before 2012 related to risk factors. Missing data presented a potential source of bias because the high likelihood the data are not missing at random. Missing values were replaced with probable values, diminishing this source of bias. We compared these results from the multiple imputed sample against the sample with only patients having all their information complete (i.e. we discarded patients with missing values) and against a sample grouping the missing values of each risk factor in a category. We obtained similar results for the adjusted model across the 3 samples. Moreover, the multiple imputed dataset achieved higher precision in the RRRs, reflected in narrower confidence intervals.

Finally, there could be a selection bias because many cases were detected in a HIV outpatient clinic. However, these cases were more susceptible to HCV infection and they often had risk practices but on the other hand, they were more closely monitored.

### Conclusion

Nosocomial and sexual transmission of acute HCV are the principal issues to address the HCV infection epidemic in our context of a large city. Quick identification of both HCV and HIV infected people and the contact trace will allow early treatment, reducing the risk of further transmission [47]. Monitoring of HIV patients will help to rule out HCV infection. Contact tracing will also allow for the identification and treatment of acute HCV cases that are not yet in contact with the health system among IDU, and to find those delaying to initiate treatment for HCV infection in a clinical setting since the time of diagnosis.

Since 2006 acute HCV infection due to nosocomial or IDU risk factors decreased. Meanwhile sexual risk factor has been increasing in men since 2012. Complete screening for sexual transmitted infections should be carried out when a person is diagnosed. MSM with sexual risk behaviours should be engaged with the health system and monitored regularly for HCV infection.

## Supporting information

S1 Table Factors associated with acute HCV infection in the period 2004–2005 compared to the period 2006–2011. **Complete case model, available case and imputed case. Barcelona city.** Hepatitis C virus, HCV; relative-risk ratio, RRR; confidence intervals, CI; injective drug user, IDU; and human immunodeficiency virus, HIV. All the models have been adjusted by sex, age, origin, educational level, type of risk factor for transmission for HCV, and HIV status. Reference categories are shown in brackets. Bold numbers highlight *P*-values<0.05 for Wald test. ^a^ At the time of HCV diagnosis. ^b^ 2013 Barcelona household income according to district where the person diagnosed of HCV lived. ^c^ Of them, 34 (60.7%) were men, 41 (73.2%) were Spanish-born, 34 (60.7%) were illiterate or had only completed primary/lower secondary studies, 41 (73.2%) lived in low/medium income districts, and none of them were HIV-infected.
(DOC)

S1 Appendix Analysis of missing values of acute cases of hepatitis C infection. Barcelona city (2004–2015).
(DOCX)

S2 Appendix Dataset. (XLS)
