# Supplementary material for: Evolution of acute hepatitis C virus infection in a large European city: Trends and new patterns
Source: PLoS One. 2017 Nov 14;12(11):e0187893. doi: 10.1371/journal.pone.0187893 (PMC5685589; doi:10.1371/journal.pone.0187893)
Supplement: S1 Table — Complete case model, available case and imputed case. Barcelona city. Hepatitis C virus, HCV; relative-risk ratio, RRR; confidence intervals, CI; injective drug user, IDU; and human immunodeficiency virus, HIV. All the models have been adjusted by sex, age, origin, educational level, type of risk factor for transmission for HCV, and HIV status. Reference categories are shown in brackets. Bold numbers highlight P-values<0.05 for Wald test. a At the time of HCV diagnosis. b 2013 Barcelona household income according to district where the person diagnosed of HCV lived. c Of them, 34 (60.7%) were men, 41 (73.2%) were Spanish-born, 34 (60.7%) were illiterate or had only completed primary/lower secondary studies, 41 (73.2%) lived in low/medium income districts, and none of them were HIV-infected. (DOC) [file pone.0187893.s001.doc]

## Title page

**Full title:** Evolution of acute hepatitis C virus infection in a large European city: trends and new patterns.

**Short title:** Acute hepatitis C trends in a large city.

**Author names:** César Garriga^1,2¶*,#a^, Sandra Manzanares-Laya^1,3¶^,Patricia García de Olalla^1,3^, Pilar Gorrindo^1^, Sabela Lens^4,12^, Ricard Solà^5^, Maria Martínez-Rebollar^6^, Montserrat Laguno^6^, Jordi Navarro^7^, Xavier Torras^8,12^, Mercè Gurguí^9^, María-Jesús Barberá^10^, Josep Quer^11^, Eva Masdeu^1^, Pere Simón^1^, Miriam Ros^1^, Anna de Andrés^1^, Joan A. Caylà^1,3^.

**Affiliation and address of the authors:**

^1^ Epidemiology Service, Public Health Agency of Barcelona, Barcelona, Spain.

^2^ Spanish Field Epidemiology Training Programme, National Centre for Epidemiology, Instituto de Salud Carlos III, Madrid, Spain.

^3^ CIBER de Epidemiología y Salud Pública (CIBERESP), Spain.

^4^ Liver Unit, Hospital Clínic, Barcelona. IDIBAPS.

^5^ Internal Medicine-Infectious Diseases, Hospital del Mar, Barcelona, Spain.

^6^ Hospital Clinic- August Pi i Sunyer Biomedical Research Institute, University of Barcelona, Barcelona, Spain.

^7^ Infectious Diseases, Hospital Vall de Hebron, Universitat Autònoma de Barcelona, Barcelona, Spain.

^8^ Department of Gastroenterology, Hospital de la Santa Creu i Sant Pau, CIBERehd, Barcelona, Spain.

^9^ Infectious Diseases Unit, Hospital de la Santa Creu i Sant Pau, Universitat Autònoma de Barcelona, Barcelona, Spain.

^10^ Sexually Transmitted Infections Unit, Hospital Vall de Hebron, Universitat Autònoma de Barcelona, Barcelona, Spain.

^11^ Liver Unit, Internal Medicine, Laboratory of Malalties Hepàtiques, Vall d'Hebron Institut de Recerca-Hospital Universitari Vall d´Hebron, (VHIR-HUVH), Universitat Autònoma de Barcelona, Barcelona, Spain.

^12^ CIBER of Hepatic and Digestive Diseases (CIBERehd), Madrid, Spain.

^¶^ These authors contributed equally to this work.

^#a^ Current Address: Nuffield Department of Orthopaedics, Rheumatology and Musculoskeletal Sciences. University of Oxford. Headington. Oxford. Oxfordshire. United Kingdom.

Corresponding author: E-mail: [cesar.garriga-fuentes@ndorms.ox.ac.uk](mailto:cesar.garriga-fuentes@ndorms.ox.ac.uk) (CG)

Alternative corresponding author: E-mail: [smanzana@aspb.cat](mailto:smanzana@aspb.cat) (SM)

**S1 Table**. Factors associated with acute HCV infection in the period 2004 – 2005 compared to the period 2006 – 2011. Complete case model, available case and imputed case. Barcelona city.

|  |  | **Complete cases model (n=121)** | | | | **Available cases model (n=204)** | | | | **Imputed model (n=204)** | | | |
| --- | --- | --- | --- | --- | --- | --- | --- | --- | --- | --- | --- | --- | --- |
|  |  | **Crude analysis** | | **Adjusted analysis** | | **Crude analysis** | | **Adjusted analysis** | | **Crude analysis** | | **Adjusted analysis** | |
| **Variables** | **Categories** | **RRR** | **95% CI** | **RRR** | **95% CI** | **RRR** | **95% CI** | **RRR** | **95% CI** | **RRR** | **95% CI** | **RRR** | **95% CI** |
| **Sex** | (Women) |  |  |  |  |  |  |  |  |  |  |  |  |
|  | Men | 0.8 | [0.3–2.8] | 0.9 | [0.2–3.2] | 0.6 | [0.3–1.2] | 0.5 | [0.2–1.2] | 0.6 | [0.3–1.2] | 0.6 | [0.2–1.6] |
| **Age^a^** | (Continuous variable, 5 years groups) | 1.1 | [0.9–1.3] | 1.0 | [0.8–1.3] | 1.0 | [0.9–1.2] | 1.0 | [0.9–1.2] | 1.0 | [0.9–1.2] | 1.0 | [0.9–1.2] |
| **Country of origin** | (Spanish–born) |  |  |  |  |  |  |  |  |  |  |  |  |
|  | Foreign born | 0.7 | [0.2–3.2] | 0.6 | [0.1–3.2] | 1.1 | [0.5–2.6] | 1.0 | [0.3–2.9] | 1.1 | [0.5–2.6] | 0.9 | [0.3–2.8] |
| **Educational level completed** | (Illiteracy/Primary/Lower secondary) |  |  |  |  |  |  |  |  |  |  |  |  |
|  | Upper secondary/University | 1.6 | [0.5–5.3] | 1.5 | [0.4–5.5] | 0.9 | [0.4–2.3] | 1.2 | [0.4–3.1] | 0.9 | [0.4–2.3] | 1.0 | [0.4–2.6] |
|  | Missing | ― | ― | ― | ― | 1.8 | [0.6–5.3] | 1.7 | [0.5–6.3] | ― | ― |  |  |
| **District household income^b^** | (Low–medium) |  |  |  |  |  |  |  |  |  |  |  |  |
|  | High | 3.6 | [0.8–16.5] |  |  | 2.3 | [0.8–6.5] |  |  | 2.4 | [0.9–6.7] |  |  |
|  | Very high | 1.9 | [0.3–13.4] |  |  | 1.0 | [0.2–4.8] |  |  | 1.0 | [0.2–4.7] |  |  |
|  | Missing | ― | ― |  |  | 4205753.0 | [0.0–.] |  |  | ― | ― |  |  |
| **Risk factor for HCV transmission** | (Nosocomial) |  |  |  |  |  |  |  |  |  |  |  |  |
|  | Sexual | 0.4 | [<0.1–4.4] | 0.4 | [<0.1–5.0] | 0.4 | [<0.1–4.2] | 0.4 | [<0.1–4.5] | 0.4 | [<0.1–4.0] | 0.4 | [<0.1–5.6] |
|  | IDU | 0.3 | [<0.1–2.3] | 0.3 | [<0.1–2.8] | 0.2 | [<0.1–1.7] | 0.2 | [<0.1–2.0] | 0.2 | [<0.1–2.3] | 0.3 | [<0.1–3.6] |
|  | Others: acupuncture, tattoo... | 3.5 | [0.3–42.8] | 5.4 | [0.4–84.2] | 5.1 | [0.5–54.0] | 6.4 | [0.6–74.6] | 5.7 | [0.6–59.6] | 7.3 | [0.5–99.0] |
|  | Missing^c^ | ― | ― |  |  | **2.8** | [1.2–6.8] | **3.0** | [1.2–7.8] | ― | ― |  |  |
| **HIV status^a^** | (Negative) |  |  |  |  |  |  |  |  |  |  |  |  |
|  | Positive | 2.1 | [0.1–36.5] | 4.4 | [0.2–92.4] | 1.1 | [<0.1–17.7] | 4.3 | [0.2–87.5] | 1.1 | [0.1–17.7] | 2.0 | [0.1–39.3] |

Hepatitis C virus, HCV; relative-risk ratio, RRR; confidence intervals, CI; injective drug user, IDU; and human immunodeficiency virus, HIV. All the models have been adjusted by sex, age, origin, educational level, type of risk factor for transmission for HCV, and HIV status. Reference categories are shown in brackets. Bold numbers highlight *P*-values<0.05 for Wald test.

^a^ At the time of HCV diagnosis.

^b^ 2013 Barcelona household income according to district where the person diagnosed of HCV lived.

^c^ Of them, 34 (60.7%) were men, 41 (73.2%) were Spanish-born, 34 (60.7%) were illiterate or had only completed primary/lower secondary studies, 41 (73.2%) lived in low/medium income districts, and none of them were HIV-infected.
