# Supplementary material for: Evolution of acute hepatitis C virus infection in a large European city: Trends and new patterns
Source: PLoS One. 2017 Nov 14;12(11):e0187893. doi: 10.1371/journal.pone.0187893 (PMC5685589; doi:10.1371/journal.pone.0187893)
Supplement: S1 Appendix — Barcelona city (2004–2015). (DOCX) [file pone.0187893.s002.docx]

**S1 Appendix. Analysis of missing values of acute cases of hepatitis C infection. Barcelona city (2004-2015).**

Missing values are suitable for imputation when they are not “missing not at random” (MNAR). Our data were not MNAR because we did not observe a monotonous pattern (Fig. A1) for loss of information in our dataset, hence our missing data are “ignorable”. Ignorable means that the data are suitable for multiple imputation. According to the pattern of missing values in our incomplete variables (district household income, educational level completed and risk factors for HCV transmission) we observed a random pattern.

**Fig. A1. Missing value patterns.**


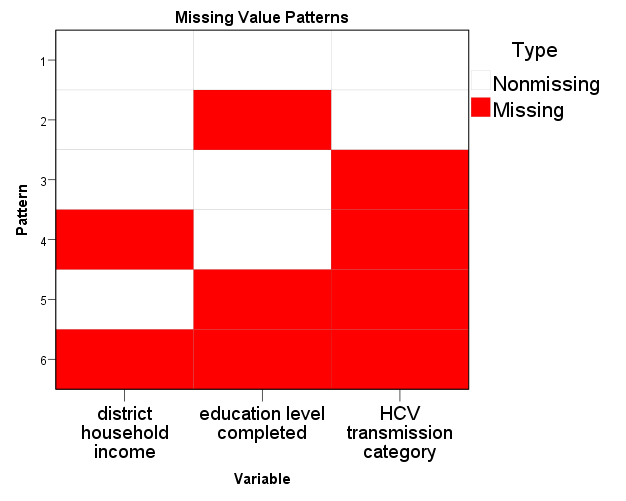


In our dataset there were 3 variables with missing values (education level completed, district household income and risk factors for HCV transmission) and four with all their information complete (sex, age, country of origin, HIV status). The outcome year of HCV had also complete data. We also checked if the data were “missing completely at random” (MCAR) or simply “missing at random”. MCAR would provide inferences without bias using “complete case analysis” while if MAR values were used in a complete case analysis biased inferences would be obtained. Table A1 shows statistically significant differences in the distribution of missing values across the categories of the risk factors. Table A2 also shows statistically significant differences between incomplete and complete cases across the categories of the risk factors. These tables show that the data are not MCAR because there is some relationship between missing values and the risk factors. Therefore, the use of multiple imputation benefits the inferences more than only using complete case analysis in order to obtain estimations without bias and not loosing cases that would reduce the statistical power of the estimation.

**Table A1. Distribution of acute cases of hepatitis C virus infection and percentage of missing values per variable. Barcelona city (2004-2015).**

|  | **n** | **%** | **% missing values** | | |
| --- | --- | --- | --- | --- | --- |
| **Total** | **204** | **100** | **Education level** | **District household income** | **risk factors for HCV transmission** |
| **Period** |  |  |  | *P^c^ =* 0.004 | *P*<0.001 |
| 2004 – 2005 | 53 | 26.0 | 20.8 | 7.6 | 66.0 |
| 2006 – 2011 | 57 | 27.9 | 12.3 | 0.0 | 36.8 |
| 2012 – 2015 | 94 | 46.1 | 19.2 | 0.0 | 5.3 |
| **Sex** |  |  |  |  | *P* = 0.013 |
| Men | 151 | 74.0 | 18.5 | 2.0 | 25.2 |
| Women | 53 | 26.0 | 15.1 | 1.9 | 43.4 |
| **Age, years^a^** |  |  | *P^c^ =* 0.009 |  |  |
| <15 | 1 | 0.5 | 0.0 | 0.0 | 0.0 |
| 15-24 | 11 | 5.4 | 36.4 | 0.0 | 18.2 |
| 25-34 | 35 | 17.2 | 28.6 | 5.7 | 25.7 |
| 35-44 | 59 | 28.9 | 23.7 | 0.0 | 25.4 |
| 45-54 | 48 | 23.5 | 10.4 | 2.1 | 22.9 |
| ≥55 | 50 | 24.5 | 6.0 | 2.0 | 48.0 |
| **Country of origin** |  |  |  |  |  |
| Spanish-born | 135 | 66.2 | 14.1 | 1.5 | 34.1 |
| Foreign born | 69 | 33.8 | 24.6 | 2.9 | 21.7 |
| **Education level completed** |  |  | *P*<0.001 | *P^c^ =* 0.018 | *P*<0.001 |
| Illiteracy/Primary/Lower secondary | 81 | 39.8 | – | 0.0 | 44.4 |
| Upper secondary/University | 87 | 42.6 | – | 1.2 | 12.6 |
| Missing | 36 | 17.6 | – | 8.3 | 38.9 |
| **District household income^a, b^** |  |  | *P^c^ =* 0.040 |  | *P^c^ =* 0.001 |
| Low-medium | 129 | 63.2 | 18.6 | – | 34.1 |
| High | 59 | 28.9 | 13.6 | – | 15.3 |
| Very high | 12 | 5.9 | 8.3 | – | 33.3 |
| Missing | 4 | 2.0 | 75.0 | – | 100.0 |
| **Risk factors for HCV transmission** |  |  |  |  |  |
| Sexual | 74 | 36.3 | 13.5 | 0.0 | – |
| Nosocomial | 48 | 23.5 | 14.6 | 0.0 | – |
| IDU | 14 | 6.9 | 21.4 | 0.0 | – |
| Others | 7 | 3.4 | 28.6 | 0.0 | – |
| Missing | 61^g^ | 29.9 | 23.0 | 6.6 | – |
| **HIV status^a^** |  |  |  |  | *P*<0.001 |
| Positive | 66 | 32.4 | 15.2 | 0.0 | 0.0 |
| Negative | 138 | 67.6 | 18.8 | 2.9 | 44.2 |

Hepatitis C virus, HCV; standard deviation, SD; injective drug user, IDU; human immunodeficiency virus, HIV

^a^ At the time of HCV diagnosis.

^b^ 2013 Barcelona household income according to district where the person diagnosed of HCV lived.

^c^ Significant level of 2-sided Fisher's exact statistic.

*P* values of univariable analysis are showed when they became statistically significant. E.g. *P*=0.013 corresponds to the row of “sex” and column of “risk factors for HCV transmission”. This *P* value means that the distribution of missing values of risk factors for HCV transmission is different in men than in women.

**Table A2. Distribution of risk factors for acute cases of hepatitis C virus infection by complete and incomplete cases. Barcelona city (2004-2015).**

|  | **Incomplete cases** | | **Complete cases** | | ***P* value** |
| --- | --- | --- | --- | --- | --- |
| **Total** | **n** | **%** | **n** | **%** |  |
| **Period** |  |  |  |  | *P*<0.001 |
| 2004 – 2005 | 37 | 44.6 | 16 | 13.2 |  |
| 2006 – 2011 | 24 | 28.9 | 33 | 27.3 |  |
| 2012 – 2015 | 22 | 26.5 | 72 | 59.5 |  |
| **Sex** |  |  |  |  | *P*= 0.14 |
| Men | 57 | 68.7 | 94 | 77.7 |  |
| Women | 26 | 31.3 | 27 | 22.3 |  |
| **Age, years^a^** |  |  |  |  | *P*= 0.49 |
| <15 | 0 | 0.0 | 1 | 0.8 |  |
| 15-24 | 4 | 4.8 | 7 | 5.8 |  |
| 25-34 | 15 | 18.1 | 20 | 16.5 |  |
| 35-44 | 24 | 28.9 | 35 | 28.9 |  |
| 45-54 | 15 | 18.1 | 33 | 27.3 |  |
| ≥55 | 25 | 30.1 | 25 | 20.7 |  |
| **Country of origin** |  |  |  |  | *P =* 0.22 |
| Spanish-born | 59 | 71.1 | 76 | 62.8 |  |
| Foreign born | 24 | 28.9 | 45 | 37.2 |  |
| **Education level completed** |  |  |  |  | *P*<0.001 |
| Illiteracy/Primary/Lower secondary | 36 | 76.6 | 45 | 37.2 |  |
| Upper secondary/University | 11 | 23.4 | 76 | 62.8 |  |
| **District household income^a, b^** |  |  |  |  | *P =* 0.07 |
| Low-medium | 58 | 73.4 | 71 | 58.7 |  |
| High | 16 | 20.3 | 43 | 35.5 |  |
| Very high | 5 | 6.3 | 7 | 5.8 |  |
| **Risk factors for HCV transmission** |  |  |  |  | *P^c^ =*0.53 |
| Sexual | 10 | 45.5 | 64 | 52.9 |  |
| Nosocomial | 7 | 31.8 | 41 | 33.9 |  |
| IDU | 3 | 13.6 | 11 | 9.1 |  |
| Others | 2 | 9.1 | 5 | 4.1 |  |
| **HIV status^a^** |  |  |  |  | *P*<0.001 |
| Positive | 10 | 12.1 | 56 | 46.3 |  |
| Negative | 73 | 88.0 | 65 | 53.7 |  |

Hepatitis C virus, HCV; standard deviation, SD; injective drug user, IDU; human immunodeficiency virus, HIV

^a^ At the time of HCV diagnosis.

^b^ 2013 Barcelona household income according to district where the person diagnosed of HCV lived.

^c^ Significant level of 2-sided Fisher's exact statistic.

P values of univariable analysis are showed when they became statistically significant.
